# The WHO Kobe Centre; three decades of contributions to public health research

**DOI:** 10.2471/BLT.25.295039

**Published:** 2025-12-01

**Authors:** Sarah L Barber

**Affiliations:** a WHO Centre for Health Development, World Health Organization, 1-5-1 Wakinohama-Kaigandori, Chuo-Ku Kobe 651-0073, Japan.

The World Health Organization (WHO) Centre for Health Development (also known as the WHO Kobe Centre) is currently a global research centre for health and long-term care systems, ageing populations and disaster preparedness. Though part of WHO headquarters, the centre operated as an outposted office in Kobe, Japan since 1996 with support from the Kobe Group and the Hyogo and Kobe communities.

Since its inception, one of the centre’s achievements has been positioning Kobe on the global public health stage, hosting international symposia and conferences on urbanization, earthquakes, women’s health and tobacco control, among others. From 1996 to 2005, the centre led the Knowledge Network on Urban Settings, which is part of the WHO Commission on the Social Determinants of Health.

Between 2006 and 2015, the centre achieved several milestones including the publication of the *Knowledge network on urban settings report*;^1^ the *Urban health equity assessment and response tool*;^2^ and the first of two WHO global reports on urban health with UN-Habitat,^3^^,^^4^ as well as leading the World Health Day campaign on Urban Health Matters, the first office outside Geneva to coordinate World Health Day. In 2012, Kobe hosted the first WHO Global Forum on Innovation for Ageing Populations, consolidating the centre’s global leadership on ageing.

From 2016 to 2025, the centre’s research focused on innovative policy approaches for sustainable universal health coverage (UHC) amid ageing demographics. Achievements in service delivery and sustainable financing included research collaborations with the Organisation for Economic Co-operation and Development on price setting and purchasing in health care and long-term care;^5^ working with the European Observatory on Health Systems and Policies to develop a simulator for decision-makers to explore policy options regarding the financial implications of ageing;^6^ and studies showing that investing in quality long-term care is a strategy for promoting economic development for countries at all levels of development.^7^

The centre researched measurement of unmet health and social care needs of older people to improve monitoring progress on UHC. Achievements included informing discussions at the World Health Assembly and United Nations High-level meeting on UHC in 2023;^8^ supporting the establishment of a global research consortium, CARETRACK, to produce evidence on unmet care needs of older people; and conducting the first cross-regional study of unmet health and social care needs.^9^

As the secretariat of the WHO Thematic Platform for Health Emergencies and Disaster Risk Management Research Network, the centre accelerated research on disaster prevention, preparedness and response. The centre also coordinated WHO’s first reference book on research methods for disaster risk management in 2021, which was updated with a chapter on the coronavirus disease 2019 (COVID-19) in 2022,^10^ translated into Japanese in 2024, and is undergoing review for the second edition. In the centre’s upcoming thirtieth anniversary report (in press),^11^ Shigeru Omi, WHO Regional Director in the Western Pacific (1998–2008) and a member of the WHO Kobe Centre’s Advisory Committee (2009–2011), who chaired the Japanese government’s panel on the COVID-19 response, praised the centre’s translation of over 300 WHO technical guidance documents during the pandemic. 

At the local level, the centre partnered with universities in the Kansai area to address domestic health research needs and benefit the community. Its largest project, the Kobe Project for Managing Dementia Patients, in collaboration with Kobe University, Kobe Municipality and the Kobe Biomedical Innovation Cluster, ran from 2017 to 2023.^12^ This project created a model for using municipal data in academic research, informing early detection and management policies for patients with dementia or cognitive decline.

Over the past decade, the centre collaborated with nearly 160 global and domestic research partners, produced over 250 publications and dozens of research reports, and leveraged its partner networks to disseminate research findings to policy-makers. The centre also supported future public health professionals through WHO’s internship programme, welcoming over 90 international students since 2006 who later developed careers that contribute to advancing public health in Japan and abroad.

Following 30 years of collaboration, the Kobe Group, a coalition of local public and private entities in Hyogo Prefecture, announced that it could no longer provide financial support. As a result, the WHO Kobe Centre will close by 31 March 2026.

WHO extends its heartfelt thanks to the Kobe Group and the citizens of Hyogo Prefecture and Kobe City for 30 years of sustained support. Their commitment enabled the centre to promote global health development and establish Kobe as a hub for scientific research and advancement. The centre’s legacy will continue through its partners and the impact of its work for future generations.
